# Ocular TB as the only manifestation of TB: diagnostic uncertainty and treatment thresholds

**DOI:** 10.1007/s10792-026-04134-3

**Published:** 2026-06-19

**Authors:** Sedra T. Alabed, Diana Finkel

**Affiliations:** Department of Infectious Diseases, Rutgers New Jersey Medical School, Newark, NJ USA

**Keywords:** Ocular tuberculosis, Tuberculous uveitis, Interferon-gamma release assay, Latent tuberculosis infection, Retinal vasculitis, Serpiginous-like choroiditis

## Abstract

**Purpose:**

To review diagnostic approaches and treatment thresholds for presumed tuberculous uveitis when ocular inflammation is the only manifestation of tuberculosis (TB), focusing on phenotype-based risk stratification, interferon-gamma release assay (IGRA) interpretation, mimic exclusion, and evidence for antitubercular therapy (ATT).

**Methods:**

Narrative review using targeted PubMed search of ocular TB literature. Search terms included “ocular tuberculosis”, “tuberculous uveitis”, specific phenotypes (serpiginous-like choroiditis, retinal vasculitis, tuberculoma), and treatment outcomes. We prioritized consensus guidelines, systematic reviews, meta-analyses, and randomized data. Findings were synthesized into a phenotype-anchored diagnostic and treatment algorithm.

**Results:**

Ocular TB is usually diagnosed through a comprehensive assessment of ocular phenotype, epidemiologic risk, systemic evaluation, TB immunologic testing, and mimic exclusion. Higher-suspicion phenotypes include serpiginous-like choroiditis, occlusive retinal vasculitis, choroidal tuberculoma, anterior uveitis with iris nodules, and chronic granulomatous anterior uveitis in the appropriate clinical context. IGRA results support prior TB sensitization but do not establish ocular causality; positive results may be incidental, particularly in low-burden settings, while negative results do not fully exclude ocular TB when phenotype and epidemiologic context are strongly suggestive. Consensus guidance and recent randomized evidence support ATT in selected patients, but treatment thresholds remain phenotype- and context-dependent.

**Conclusion:**

Compatible ocular phenotypes should prompt TB-directed evaluation, including IGRA and systemic assessment, with immunologic testing interpreted as supportive evidence. Treatment decisions should consider phenotype, epidemiological risk, mimic exclusion, and consequences of delayed treatment. High-risk presentations may justify lower treatment thresholds, while nonspecific ocular findings with isolated immunologic positivity should prompt consideration of alternative diagnoses or LTBI management according to local guidance.

## Introduction

Tuberculosis (TB) is an infectious disease caused by *Mycobacterium tuberculosis*, an acid-fast bacillus, that primarily causes pulmonary disease but can also lead to extrapulmonary manifestations [1, 2]. Ocular TB is one of the rare but critical extrapulmonary manifestations of the disease that can lead to potential vision loss [3, 4]. It may involve any part of the ocular tissue and can manifest without a prior history of pulmonary TB [5].

Most commonly, ocular TB presents as uveitis and is estimated to account for about 4.0% of uveitis cases globally, with higher rates in high-burden countries and sub-Saharan Africa [3, 6–9]. The reported prevalence remains variable due to the difficulty of making an accurate diagnosis of tuberculous uveitis [10, 11]. Uveitis has a broad differential diagnosis, with etiologies generally categorized as infectious or immune-mediated, and many cases remaining idiopathic after evaluation [12–14]. The various potential variables and lack of consensus on diagnostic approaches render the diagnosis of uveitis complex.

In tuberculous uveitis, the diagnosis is often made clinically supported by indirect evidence of TB and a compatible ocular phenotype [15]. This contrasts with pulmonary TB, where the diagnosis is typically microbiologically confirmed. Routinely, intraocular sampling is avoided due to impracticality, low-yield secondary to organisms being sequestered in tissue rather than free fluid or prior antimicrobial exposure, and frequent false negatives [16, 17]. Instead, patients are referred for treatment based on ophthalmic exam findings and a positive interferon-gamma release assay (IGRA), even in the absence of systemic symptoms [18]. The use of IGRA poses a limitation, as it is a test that measures host immune sensitization to *Mycobacterium tuberculosis* antigens, thus providing evidence of TB infection at some point without distinguishing active from latent TB infection [19, 20]. This means that IGRA may be positive in individuals whose uveitis is not related to TB. Such diagnostic uncertainty can lead to different schools of thought when approaching treatment. Importantly, even when LTBI prophylaxis is utilized, recent evidence suggests it reduces but does not eliminate TB risk, underscoring the need for ongoing surveillance [21]. While some clinicians might favor initiating antitubercular therapy (ATT), others may favor immunosuppression when there is a lack of systemic evidence. The typical ATT recommendation by the World Health Organization is a 6-month regimen (isoniazid/rifampicin/pyrazinamide/ethambutol for 2 months, followed by isoniazid/rifampicin for 4 months).

To address this dilemma, consensus guidelines were proposed by experts as part of the Collaborative Ocular Tuberculosis Study (COTS) [22]. The guidelines were created using clinical expert evidence, as well as data in the available literature, which resulted in recommendations for initiating ATT based on the phenotype of uveitis such as choroiditis/serpiginous-like choroiditis, retinal vasculitis, or pan uveitis [22]. Despite such efforts to standardize the treatment of ocular TB, there are still ongoing disagreements that emphasize the largely observational basis of treatment, with variable definitions, endemicity, adjunctive immunosuppression therapies, and outcome measures [10, 15, 23–25]. This review synthesizes current literature into a phenotype-based framework for interpreting IGRA/TST results, excluding mimics, evaluating for systemic TB, and deciding when ATT is justified in the absence of microbiologic confirmation or active systemic disease.

## Methods

This narrative review was conducted using the literature on the diagnosis and management of ocular TB when uveitis is the only or predominant manifestation of TB. A targeted search of PubMed/MEDLINE was performed with a focus on the definitions and classification frameworks for ocular TB, the interpretation of IGRA/TST in ocular TB, the phenotype-based approaches and multimodal imaging findings, as well as the recommended systemic evaluations for mimic exclusion, and treatment outcomes with ATT with or without adjunctive corticosteroids/immunosuppression. Search terms included combinations of “ocular tuberculosis,” “tuberculous uveitis,” “interferon-gamma release assay,” “QuantiFERON,” “serpiginous-like choroiditis,” “retinal vasculitis,” “Eales,” “tuberculoma,” “COTS,” and “treatment outcomes.” Eligible publications were English-language articles from January 2000 through March 2026, with priority given to those from the past ten years. Literature search was conducted between January 2026 until March 2026. Reference lists of included guidelines and reviews were hand-searched for additional studies. Consensus statements, systematic reviews, meta-analyses, randomized controlled trials, prospective cohorts, and large retrospective series were included. Case reports, conference abstracts, and non-peer-reviewed sources were excluded unless they uniquely illustrated a phenotype or decision point. As a narrative review, no formal screening protocol, blinded dual review, risk-of-bias assessment, or quantitative synthesis was performed. Evidence was evaluated qualitatively with emphasis on practical decision points, such as pre-test probability (endemicity and epidemiologic risk), phenotype specificity, systemic workup, and exclusion of mimics.

The evidence base for ocular TB is dominated by observational, single-center, and registry data with heterogeneous diagnostic definitions and outcome measures, and conclusions in this review are best regarded as evidence-informed clinical guidance rather than the product of a systematic synthesis [25, 26].

## Definitions and diagnostic framework

Ocular TB is typically classified by diagnostic uncertainty rather than by a single definitive test. The term “confirmed ocular TB” is used when referring to ocular inflammation on ophthalmic examination with microbiological or histopathological evidence of *Mycobacterium tuberculosis* from an intraocular tissue or fluid sample. On the other hand, “presumed ocular TB” is used in reference to cases of uveitis in the setting of TB infection evidence via IGRA, tuberculin skin test (TST), and/or supportive systemic evaluation with no better alternative etiological explanation. This framework is cited across various literature, with an explicit acknowledgement of the lack of gold standard resulting in variability in reported incidence and outcomes [15, 22, 25–27]. Another approach by the Standardization of Uveitis Nomenclature (SUN) Working Group provided a classification criteria for research standardization to help improve comparability across studies. The criteria define tuberculous uveitis as a syndrome based on characteristic phenotypes, such as serpiginous-like choroiditis, occlusive retinal vasculitis, choroidal granuloma, and anterior uveitis with iris nodules, in addition to evidence of TB infection [26].

Evidence of TB infection is routinely assessed via IGRA or TST. Intraocular sampling may be considered in some cases, but culture and nucleic-acid amplification testing (PCR) have limited sensitivity because ocular TB is typically paucibacillary and organisms may be sequestered in the tissue and not freely circulating in the aqueous or vitreous humor, raising the risk of false-negatives [16, 28]. A positive ocular PCR can support the diagnosis, but a negative result does not exclude it [29].

IGRA and TST indicate immune sensitization to *Mycobacterium tuberculosis* but do not distinguish latent infection from active ocular disease. The distinction of active versus latent TB becomes important in cases of uveitis because the pre-test probability that TB is the cause of inflammation varies by phenotype, as well as epidemiologic context such as endemicity [30, 31]. The diagnostic value of IGRA in low-prevalence settings hinges on whether the ocular phenotype is itself suggestive of TB. In a recent series from a low-endemic U.S. center, the positive predictive value of QuantiFERON-Gold for tubercular uveitis was 100% when SUN classification criteria were met but 0% when they were not, and most positives were incidental rather than disease-attributable [32]. The opposite applies in endemic settings, where high background prevalence of latent infection means a positive IGRA contributes only modestly to the post-test probability that uveitis is tuberculous; in those populations, the result is more usefully weighted alongside phenotype specificity and a structured mimic exclusion than treated as a stand-alone confirmatory test [33–35].

**Fig. 1:**
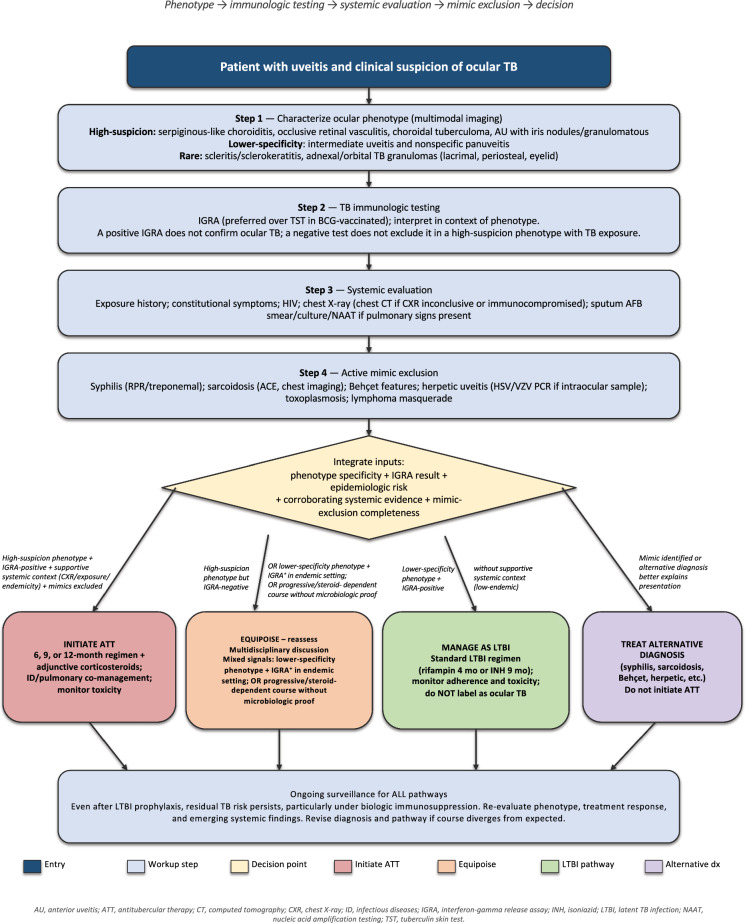
Sequential diagnostic and treatment algorithm for presumed ocular tuberculosis

Additionally, a negative IGRA should not automatically exclude ocular TB, especially in high-risk phenotypes in TB-endemic regions. A negative test may reflect paucibacillary disease in which the host immune system fails to mount a detectable interferon-gamma response, iatrogenic or disease-related immunosuppression, or true absence of TB infection. Negative testing should also force reconsideration of non-TB inflammatory chorioretinopathies and other mimics. When the ocular phenotype is highly compatible, disease is vision-threatening, and alternatives have been carefully excluded, treatment may still be justified after multidisciplinary discussion, even without IGRA/TST positivity [19, 30, 34]. Figure 1 summarizes a phenotype-first approach to presumed ocular TB. In this framework, IGRA/TST results are interpreted as supportive evidence rather than as stand-alone diagnostic tests, and treatment thresholds are adjusted according to phenotype specificity, epidemiologic risk, systemic findings, mimic exclusion, and the consequences of delayed therapy.

The algorithm outlines a stepwise approach to patients with uveitis where ocular TB is being considered regardless of IGRA status. Initial evaluation begins with characterization of the ocular phenotype, followed by TB immunologic testing, systemic evaluation for active or prior TB, and exclusion of infectious, inflammatory, and masquerade mimics. The final treatment pathway depends on the combined weight of phenotype specificity, IGRA/TST results, epidemiologic risk, corroborating systemic findings, and completeness of mimic exclusion. High-suspicion phenotypes with supportive TB evidence may justify antitubercular therapy, whereas lower-specificity phenotypes with isolated immunologic positivity may be better managed as latent TB infection according to local guidelines or an alternative diagnosis. All pathways require continued follow-up, particularly in patients receiving immunosuppression. AU, anterior uveitis; ATT, antitubercular therapy; CT, computed tomography; CXR, chest X-ray; ID, infectious diseases; IGRA, interferon-gamma release assay; INH, isoniazid; LTBI, latent tuberculosis infection; NAAT, nucleic acid amplification testing; TB, tuberculosis; TST, tuberculin skin test.

## Ocular phenotypes associated with TB and treatment-threshold implications

The clinical diagnosis of ocular TB often factors the phenotypic presentation at ophthalmic examination. To standardize research criteria for tuberculous uveitis and create unifying language, the SUN Working Group described a set of compatible uveitis syndromes. Certain posterior segment syndromes tend to occur more commonly across different groups, while other non-specific anterior or intermediate patterns are less common and have broader differentials with low specificity [26]. This approach emphasizes the current best approach to ocular TB, where in the absence of microbiological confirmation, clinicians have leaned towards recognizable clinical patterns alongside the evidence of TB infection. The ability to identify these patterns is via multimodal imaging modalities, such as fundus autofluorescence, optical coherence tomography (OCT), fluorescein angiography, indocyanine green angiography, and OCT angiography.

### High-suspicion posterior phenotypes

Serpiginous-like tubercular choroiditis is a distinct posterior uveitis phenotype that presents with inflammation of the choriocapillaris and the retinal pigment epithelium. Lesions may be multifocal (discrete or confluent) or placoid with actively inflamed edges but healed centers extending beyond the peripapillary region in a serpiginoid pattern [36, 37]. A key point is distinguishing this phenotype from idiopathic serpiginous choroiditis or primary inflammatory choriocapillaropathies. The overall pattern becomes critical, where imaging findings should be assessed in conjunction to the epidemiologic TB risk and supportive TB testing. The specific imaging modalities used include fundus autofluorescence for detecting active edges versus old scars, OCT for assessing the involvement of retinal pigment epithelium and outer retina, and fluorescein angiography and indocyanine green angiograph to assess retinal circulation and choroidal involvement, as summarized in Table 1 [36–38]. The threshold for ATT is often lower when findings are progressive, bilateral, or steroid-dependent [26, 38].

**Table 1:** Phenotype presentations for ocular tuberculosis: typical patterns, practical multimodal imaging, key mimics, and clinical situations where ATT is more often considered in the absence of microbiologic confirmation

Phenotype	Typical clinical pattern	Imaging most useful in practice	Key mimics to exclude	When ATT is more often considered	Key refs
Serpiginous-like tubercular choroiditis	Posterior uveitis involving choriocapillaris/RPE. Lesions may be multifocal (discrete or confluent) or placoid with an actively inflamed edge and healed center; may extend beyond the peripapillary region and progress in a serpiginoid pattern over time	FAF to distinguish active edges vs scars; OCT to assess RPE/outer retina involvement; FA/ICGA to evaluate retinal circulation and choroidal involvement; OCT-A increasingly used to assess choriocapillaris flow defects	Idiopathic serpiginous choroiditis; primary inflammatory choriocapillaropathies	Progressive lesions; bilateral disease; steroid dependence (in conjunction with epidemiologic TB risk and supportive TB testing)	[26, 36–38]
Occlusive retinal vasculitis (TB-associated/“Eales spectrum”)	Retinal vasculitis with ischemic changes on angiography; may be complicated by retinal neovascularization and/or vitreous hemorrhage. Definitions vary across studies (some require occlusion/ischemia; others accept peripheral periphlebitis with positive TB immunologic testing)	Widefield FA to localize vascular leakage, map peripheral capillary hypoperfusion/nonperfusion, and detect neovascularization; angiographic pattern recognition (venous vs arteriolar predominance, degree of ischemia/occlusion, leakage pattern) combined with systemic evaluation/testing	Eales disease; Behçet disease; sarcoidosis; syphilis	Severe ischemic/occlusive angiographic disease; recurrent vitreous hemorrhage or neovascularization; recurrent activity or steroid dependence after comprehensive evaluation for mimics	[26, 39–43, 45–47]
Choroidal tuberculoma (choroidal nodule)	Discrete mass-like choroidal lesion; may be the only ocular clue to TB and can occur with minimal systemic symptoms or with active pulmonary disease. Suspicion is intermediate because the lesion is not specific to TB	OCT to characterize lesion configuration and associated RPE changes; FA to evaluate lesion perfusion and surrounding inflammatory activity; OCT-A for non-invasive microvascular follow-up; B-scan ultrasound to confirm a choroidal lesion when media opacity limits visualization and to help differentiate from intraocular tumors based on reflectivity traits	Sarcoid choroidal granuloma; intraocular lymphoma/other infiltrative masquerade processes	Greater concern when lesion behavior is progressive and/or when systemic context supports TB after exclusion of infiltrative masquerade and granulomatous mimics	[26, 48–50, 53]
Granulomatous anterior uveitis	Granulomatous anterior uveitis ± iris nodules. Primarily clinical phenotype	Slit-lamp biomicroscopy (granulomatous keratic precipitates, iris nodules, posterior synechiae). Anterior segment OCT may characterize iris nodule architecture. Gonioscopy if angle involvement suspected	Sarcoidosis, syphilis, herpetic anterior uveitis when clinically suggested	Chronic/recurrent/steroid-dependent course; epidemiologic risk for TB; positive IGRA/TST; no better alternative explanation after mimic exclusion. ATT may be considered even with negative IGRA if clinical suspicion is high	[15, 26]
Lower-specificity patterns (intermediate uveitis and nonspecific panuveitis)	Nonspecific patterns with broad differentials and low specificity for TB; intermediate uveitis TB is typically considered a diagnosis of exclusion	Imaging generally informs complications rather than etiology and is less specific than in posterior phenotypes	Noninfectious: sarcoidosis, Behçet disease. Infectious: syphilis, viral anterior uveitis/retinitis, toxoplasmosis in retinitis-like posterior presentations	IGRA/TST alone is not considered confirmatory, particularly in low-endemic settings; clinicians typically require stronger corroboration (epidemiologic risk, consistent imaging/systemic context, steroid dependence/recurrence, and negative evaluation for mimics) before initiating ATT	[26, 51, 54–57]
Scleritis / sclerokeratitis	Anterior/posterior scleritis ± scleral nodules; interstitial keratitis	Slit-lamp biomicroscopy for anterior scleritis; B-scan ultrasonography for posterior scleritis (scleral thickening, 'T-sign'); anterior segment OCT may characterize scleral nodule architecture. CT/MRI orbit if posterior extension or scleral abscess suspected	Autoimmune scleritis; GPA; syphilis; herpes zoster; sarcoidosis; malignancy	Persistent or necrotizing course unresponsive to conventional anti-inflammatory therapy; epidemiologic risk; positive IGRA/TST; common autoimmune and infectious causes excluded. Biopsy may be needed for confirmation	[58, 59]
Adnexal/orbital TB granulomas	Orbital masses; lacrimal gland/sac involvement; eyelid/periorbital cutaneous TB	CT orbit for bony destruction, abscess cavities, and soft tissue extent. MRI orbit for soft tissue characterization and optic nerve compression. B-scan ultrasonography if intraocular extension suspected. PET-CT may identify additional systemic sites for sampling	Lymphoma; sarcoidosis; nontuberculous mycobacterial infection; fungal infection (aspergillosis); idiopathic orbital inflammatory disease; IgG4-related disease	Biopsy showing caseating granulomas or positive AFB/PCR; epidemiologic risk; common causes excluded. Orbital biopsy often required to exclude malignancy before committing to ATT	[60]

Occlusive tubercular retinal vasculitis typically presents with ischemic changes on angiography and can be complicated by retinal neovascularization or vitreous hemorrhage. The definition remains variable, with some studies requiring occlusion or ischemia, while others accept peripheral periphlebitis with positive TB immunologic testing [26, 39]. The presentation largely overlaps with Eales disease, an idiopathic peripheral retinal periphlebitis prevalent in young males characterized by overlapping phases of inflammation, capillary nonperfusion, and neovascularization with vitreous hemorrhage [40, 41]. The relationship between Eales disease and TB exposure is still unclear. Some studies have summarized that many patients with Eales disease are also IGRA/TST positive or carry a high epidemiologic TB risk, which has raised the question of whether it is a TB-associated hypersensitivity or if it is caused by direct ocular infection [40–42]. Other overlapping vasculitides are Behçet disease, sarcoidosis, and syphilis [43]. Imaging-wise, widefield fluorescein angiography is used to localize active vascular leakage, map peripheral capillary hypoperfusion, as well as detect neovascularization [44, 45]. Based on the findings, treatment decisions can then be made to prioritize anti-inflammation, panretinal photocoagulation, anti-VEGF, or ATT [44–47].

Choroidal tuberculoma is a discrete mass-like lesion that may be the only presenting ocular clue for TB infection [26, 48, 49]. It is not specific to TB and can be mimicked by a sarcoid choroidal granuloma or other infiltrative processes such as intraocular lymphoma. Tuberculomas are more often solitary, larger, more intensely yellow, with full thickness choroidal involvement and vascularization, while sarcoid granulomas tend to be multiple with different choroidal layer involvement [50]. A B-scan ultrasound can help confirm the choroidal lesion when inflammation limits fundus visualization and can aid in differentiating it from intraocular tumors by differing reflectivity traits [51, 52]. Further imaging characteristics are detailed in Table 1 [49, 52, 53].

### Anterior and panuveitis phenotypes

More phenotypes associated with TB include granulomatous anterior uveitis, intermediate uveitis, and nonspecific panuveitis [26]. These phenotypical patterns are more likely to have broader differentials and weaker specificity to TB due to being shared by numerous non-infectious (e.g., sarcoidosis and Behçet’s disease) and infectious (e.g., syphilis and viral anterior uveitis) etiologies when clinical features support those diagnoses [54, 55]. Anterior uveitis with iris nodules is described by SUN Working Group as a compatible phenotype that may occur in active systemic TB and carries more weight than non-specific granulomatous inflammation without nodular lesions [26]. However, chronic granulomatous anterior uveitis without iris nodules is also considered by many clinicians and centers to be compatible with ocular TB. BTS guidance specifically states that patients with chronic granulomatous anterior uveitis typical of ocular TB with high clinical or epidemiologic suspicions should be managed with ATT and topical corticosteroids, regardless of IGRA status [15]. Iris nodules increase specificity, but their absence does not exclude a tuberculous etiology, particularly when inflammation is chronic, recurrent, or steroid-dependent in a patient with epidemiologic risk.

Intermediate uveitis and panuveitis may occasionally represent ocular TB but have a broad differential and low specificity, where tuberculous etiology would be a diagnosis of exclusion and a positive IGRA alone is not confirmatory [54–57].

### Rare ocular, scleral, and adnexal presentations

Less common presentations may involve the sclera, ocular surface, orbit, lacrimal system, or adnexa. Persistent or necrotizing scleritis, sclerokeratitis, lacrimal gland or sac disease, eyelid/periorbital cutaneous TB, and orbital inflammatory masses have been described. These presentations are less frequent than intraocular uveitic phenotypes and often require biopsy or systemic sampling to exclude malignancy, sarcoidosis, nontuberculous mycobacterial infection, or other mimics [58–60].

## Diagnostic approach when systemic TB is absent

### Systemic evaluation

In cases of suspected ocular TB, systemic evaluation typically includes screening for constitutional symptoms associated with TB, such as fever, night sweats, weight loss, or cough, as well as assessment of TB exposure risk factors including any travel or residence in an endemic region, known TB contacts, or immunosuppression [16, 61]. Chest X-ray (CXR) is the recommended initial imaging modality in suspected TB. Chest computed tomography (CT) is reserved for cases where the CXR is inconclusive, including immunocompromised patients with subtle parenchymal disease [62]. Findings that raise suspicion for active or prior TB on CXR include upper-lobe fibronodular opacities, cavitation, miliary micronodules, and lobar consolidation with mediastinal or hilar lymphadenopathy; healed disease may appear as apical scarring or calcified granulomas. Chest CT improves detection of centrilobular and tree-in-bud nodules, small cavities, and necrotic low-attenuation lymph nodes, and is particularly useful when the CXR is normal but clinical suspicion remains high [63].

Distinguishing TB from sarcoidosis on systemic imaging is often difficult, as both can produce intrathoracic lymphadenopathy and granulomatous uveitis. Bilaterally symmetric, non-necrotic hilar and right paratracheal lymphadenopathy without parenchymal disease favors sarcoidosis, especially when accompanied by elevated serum angiotensin-converting enzyme [52, 64]. Asymmetric or unilateral lymphadenopathy with central low attenuation and rim enhancement, upper-lobe cavitary or tree-in-bud disease, and microbiologic confirmation by AFB smear, culture, or nucleic-acid amplification favor TB; caseating granulomas on tissue biopsy further support the diagnosis [65]. Because granulomas in sarcoidosis can rarely show necrosis and TB granulomas are not always caseating, neither imaging nor histopathology is fully discriminatory in isolation, and integration with the ocular phenotype and microbiologic testing remains essential [62, 66].

Ocular TB can still occur in non-endemic areas and cannot be ruled out by negative chest imaging alone, with more than half of patients with extrapulmonary TB may not present with pulmonary disease [17, 64]. One study from a low-endemic region demonstrated poor diagnostic accuracy using chest radiography for ocular TB (54.5%) with a low sensitivity of 14.7% but a high specificity of 94.3% [66]. Sputum testing using acid-fast bacilli smear, nucleic acid amplification testing, and culture should be considered in cases of pulmonary symptoms or when chest imaging is suggestive of active TB. HIV testing should also be considered, especially in a high-burden area [67].

### Mimics to exclude

The presentation of ocular TB can overlap with various infectious and noninfectious inflammatory disease processes, which calls for a workup to exclude mimics before initiating ATT. For instance, syphilis testing is recommended in all uveitis patients, as ocular inflammation occurs in almost 1% of U.S. syphilis cases [17, 61]. Beyond syphilis, the mimic work up should be tailored to the presenting phenotype.

For serpiginous-like choroiditis, key mimics include idiopathic serpiginous choroiditis, APMPPE-spectrum disease, syphilis, sarcoidosis, and viral necrotizing retinitis depending on appearance. For occlusive retinal vasculitis, the differential includes Behçet disease, sarcoidosis, syphilis, lupus, and ANCA-associated vasculitis [17, 61, 68]. For tubercular choroidal nodules, the primary mimics are sarcoid choroidal granuloma, intraocular lymphoma, fungal infection, and metastatic disease. Tuberculomas tend to be solitary, larger (mean 16.01 mm versus 2.7 mm for sarcoid granulomas), intensely yellow, include full-thickness choroidal involvement, and vascularization [53]. Specifically, granulomas larger than 6.45 mm have a high discriminatory value [53]. For granulomatous anterior uveitis, potential mimickers include sarcoidosis, syphilis, viral anterior uveitis such as varicella zoster virus or herpes simplex virus when clinical features support these diagnoses; ocular toxoplasmosis and herpetic retinitis are usually clinically distinguishable from phenotype-defined ocular TB, but may still be included in the broader differential for posterior uveitis or retinitis-like presentations [17, 61]. For scleritis or adnexal/orbital disease, autoimmune scleritis, granulomatosis with polyangiitis, malignancy, and nontuberculous mycobacterial or fungal infection should be considered [58–60]. This comprehensive exclusion of mimics helps reduce the risk of misclassification and unnecessary ATT exposure, especially in low-endemic areas.

## Treatment thresholds in presumed ocular TB

### When ATT or LTBI management is favored

ATT is more likely to be justified when the following factors converge: a compatible high-suspicion phenotype (particularly posterior segment disease), active, progressive, recurrent, or steroid-dependent inflammation, epidemiologic risk for TB, supportive IGRA/TST or systemic findings, careful exclusion of mimics, and vision-threatening disease or planned immunosuppression that would be risky if TB were the underlying cause. The threshold for ATT may also be lower when the consequences of delayed treatment risk vision loss, such as progressive posterior segment inflammation, macular or optic nerve threatening disease, or when one eye has already sustained severe inflammatory damage and the fellow eye is at risk. ATT may not be justified when the ocular phenotype is nonspecific, there is no active inflammation, a better alternative explanation exists, IGRA positivity is isolated without compatible phenotype, or ATT toxicity risk is high relative to benefit. In such cases, clinicians should avoid labeling the ocular disease as TB-associated solely on the basis of immunologic testing; if active TB is excluded, LTBI management should be separated from ocular TB-directed ATT and guided by local or national recommendations, especially when immunosuppression is planned.

### How consensus and randomized evidence inform clinical judgment

The COTS consensus guidelines used a two-step Delphi method involving 81 international uveitis experts who evaluated 486 clinical scenario-based questions about when ATT is recommended in common tubercular uveitis phenotypes [69]. The COTS calculator was created, which incorporated various variables such as endemicity, clinical phenotype, and different TB tests including IGRA and TST, as well as chest radiography (https://www.oculartb.net/cots-calc). The parameters used in the calculator are phenotype, endemicity, TST result, IGRA result, and findings on chest imaging. The COTS calculator was used in a validation study with 492 patients; it showed that a COTS score of greater than or equal to 4 was more specific than clinician judgement alone for the initiation of ATT (64.3% versus 29.6%), but clinician judgement was more sensitive (95.5% versus 48.8%) [70]. The endemicity of the region impacted the calculator’s positive predictive value and sensitivity, where both were high in endemic areas compared to non-endemic ones [70].

Systematic reviews consistently demonstrate that the available literature is not homogenous and rather observational, with variable definitions in diagnosis parameters, endemicity consideration, the ATT regimens used and durations, as well as if adjunctive corticosteroid and immunosuppression is used [9, 23, 71]. A recent meta-analysis in 2022 used 49 studies which included 4,017 patients with tuberculous uveitis; a total of 83% achieved complete resolution of inflammation using ATT with adjunctive corticosteroid therapy, and about 65% reported improved visual acuity [9]. The recurrence rate of inflammation was 13% and corticosteroid therapy was tapered to the starting dose with an inflammatory flare in about 91% of patients [9, 72]. Due to the lack of control group analysis, it is important to be cautious when interpreting favorable outcomes as improvement may reflect natural history or corticosteroid effect rather than ATT specifically. In a retrospective multinational cohort study involving 801 patients treated with ATT, the reported failure rate was 12.7%, with an increased risk in cases of panuveitis and choroidal involvement [73]. The lack of standardized recruitment in most studies is a possible limitation that continues to cause variation in treatment thresholds [9, 16, 74].

The first randomized trial was published recently in 2025, where a phase 2 randomized controlled trial was conducted in Jakarta, Indonesia [75]. It is in a TB endemic country and evaluated 76 adults with newly diagnosed and active uveitis with a positive IGRA. The participants received systemic corticosteroids with or without 6 months of ATT. At the end of the 6-month period, complete resolution of inflammation occurred more frequently in the ATT plus corticosteroid group than in the corticosteroid-alone group (81.1% vs 51.3%), and relapse was less frequent in the ATT plus corticosteroid group (5.9% vs 29.2%) [75].

### Treatment duration and regimen considerations

Although standard drug-susceptible TB regimens are based on a 6-month course, ocular TB treatment duration varies across centers. BTS guidance recommends at least 6 months of ATT and notes that 9–12 months may be considered, particularly when ocular disease is severe initially or responds slowly [15, 76]. The evidence base for extended duration is derived from expert opinion and institutional experience rather than randomized data. Clinicians should consider disease severity, treatment response, and relapse risk when determining duration through multidisciplinary discussion.

### LTBI prophylaxis and breakthrough infections

While isoniazid prophylaxis is recommended for patients with LTBI who are initiating immunosuppressive therapy, recent evidence highlights important limitations. A single-center retrospective cohort study of 188 patients with uveitis who were eligible for adalimumab therapy assessed patterns in missed LTBI cases and screening results [21]. During follow-up, 12 patients (5.9%) demonstrated LTBI indicator conversion (11 PPD, 1 IGRA), prompting additional isoniazid in 11 individuals, most commonly after PPD conversion. Active TB developed in 3 patients between 10 and 21 months of adalimumab therapy, including 2 cases of pleural TB despite documented completion of a 9-month isoniazid regimen and 1 lymphadenitis case without prior prophylaxis. These findings suggest that isoniazid prophylaxis reduces but does not eliminate the risk for TB and repeat or risk adapted LTBI screening may help identify previously overlooked infections. This evidence supports the need for ongoing surveillance even in patients who have completed appropriate prophylaxis, particularly those receiving biologic immunosuppression.

## Discussion

This review highlights a practical problem in ocular TB where neither a positive nor a negative IGRA/TST result is sufficient to determine whether TB is responsible for ocular inflammation. The diagnostic threshold depends first on the ocular phenotype, then on epidemiologic risk, systemic evaluation, and the completeness of mimic exclusion. Treatment thresholds must also account for disease trajectory, visual risk, anticipated immunosuppression, and the toxicity of ATT. Figure 1 translates these factors into a threshold-based framework.

The major limitation of the field is that these thresholds remain imperfectly validated. In practical terms, phenotype modifies the treatment threshold by changing how much additional corroboration is required before ATT is reasonable. High-specificity phenotypes should lower the threshold for ATT when TB immunologic testing is positive and mimics have been actively excluded, particularly if disease is progressive, bilateral, recurrent, or steroid-dependent. Intermediate or less specific presentations should raise the threshold and generally require stronger corroboration, such as supportive systemic imaging, epidemiologic risk, compatible multimodal imaging, recurrence despite standard therapy, or absence of a more plausible alternative diagnosis. Therefore, IGRA/TST positivity is not weighted equally across phenotypes: the same positive result may support ATT in a high-risk phenotype but may be managed as LTBI in a low-specificity presentation.

Negative IGRA should not automatically exclude ocular TB when the phenotype is highly suggestive. In such cases, clinicians must weigh whether the presentation represents an immune-mediated uveitis unrelated to TB, a paucibacillary TB infection below the detection threshold of current immunologic assays, or false-negative testing due to immunosuppression. When the ocular phenotype is highly compatible, disease is vision-threatening, and alternatives have been carefully excluded, treatment may still be justified after multidisciplinary discussion [19, 30, 34].

On the outcomes side, the most meaningful recent advance is randomized evidence. The Jakarta randomized trial provides important prospective support for ATT in IGRA-positive uveitis of undetermined cause, but its implications should be interpreted through the lens of pre-test probability [75]. In particular, generalizability beyond the trial setting is also limited. In Jakarta’s high-burden context, the prior probability that a positive IGRA reflects active rather than remote infection is meaningfully higher than in low-incidence regions, where most IGRA positives in uveitis populations are incidental [32]. Trial enrollment was also based on uveitis of undetermined cause rather than the phenotype-defined tubercular uveitis syndromes that anchor most treatment decisions in clinical practice, leaving uncertainty about whether the same effect size applies to serpiginous-like choroiditis, occlusive retinal vasculitis, or choroidal tuberculoma. Outcomes were reported at six months without a placebo-controlled comparator, and a short-term immunomodulatory contribution from rifampin cannot be excluded from any antimicrobial effect [25, 75]. The authors appropriately call for multicenter studies across diverse geographic settings, including low-endemic regions, to strengthen generalizability and power. Even with those limitations, the trial provides a practical foundation for decision-making in endemic settings, where the potential benefit may reasonably outweigh the risks of a 6-month ATT regimen [75].

Recognition of when ATT is justified must be balanced against the risks of overtreatment. Symptomatic hepatitis with combined isoniazid and rifampin has been estimated at approximately 2.5% in meta-analytic data and is the principal toxicity that limits therapy [77]. Ethambutol-related optic neuropathy is uncommon at the standard 15 mg/kg/day dose (under 1%) but is dose- and duration-dependent and carries the particular concern of vision loss in patients receiving therapy directed at vision preservation [78]. Isoniazid-related peripheral neuropathy, pyrazinamide-induced hepatitis and arthralgia, and rifampin-mediated CYP450 drug interactions add to the cumulative burden, and a 6-month course imposes substantial monitoring and adherence demands. In patients without confirmed ocular TB, these risks may exceed the benefit, particularly when an alternative inflammatory or infectious diagnosis is more likely, because unnecessary ATT may delay disease-directed therapy. The threshold for initiating ATT should accordingly be higher in low-endemic settings, in older or multimorbid patients, and in lower-specificity ocular phenotypes [22, 23, 69].

The size of the “presumed” or “undifferentiated” ocular TB category likely reflects not only disease biology but also differences in diagnostic evaluation. Published studies use heterogeneous definitions, workups, and treatment thresholds, which makes comparison across settings difficult. Access to multimodal imaging, systemic testing, microbiologic evaluation, and subspecialty expertise may influence how confidently mimics are excluded before a TB attribution is made. Therefore, variation in reported outcomes may partly reflect differences in evaluation and classification, rather than differences in ocular TB biology alone [22, 25].

Taken together, these data support a risk-calibrated approach: treatment decisions should be determined based on phenotype, disease trajectory (progressive, recurrent, steroid-dependent), epidemiologic risk, IGRA/TST status, completeness of the mimic evaluation, and the consequences of delayed treatment, especially when inflammation threatens the macula, optic nerve, or fellow eye after unilateral damage. Multidisciplinary management between ophthalmology, infectious diseases, and pulmonary medicine is critical because ATT requires toxicity monitoring, interaction checks, and a risk–benefit conversation that extends beyond the eye.

## Limitations

This review has several limitations. First, it is a narrative review rather than a systematic review; therefore, no formal screening protocol, blinded dual review, risk-of-bias assessment, or quantitative synthesis was performed. Second, the ocular TB literature remains limited by heterogeneous diagnostic definitions, variable endemicity, inconsistent systemic workup, and differences in ATT regimens, corticosteroid use, and outcome measures across studies. Third, microbiologic confirmation of ocular TB is uncommon, so much of the available evidence relies on presumed disease definitions that may over- or under-attribute ocular inflammation to TB depending on local prevalence and diagnostic resources. Finally, although recent randomized evidence supports ATT in IGRA-positive uveitis of undetermined cause in an endemic setting, its generalizability to low-burden regions and to specific phenotype-defined ocular TB syndromes remains uncertain.

## Conclusion

When ocular inflammation is the only manifestation of TB, diagnosis is usually clinical and depends on phenotype, pretest probability, as well as exclusion of mimics, with IGRA/TST used as supportive evidence rather than culture or tissue driven diagnosis. Posterior segment patterns that are strongly associated with TB, especially if recurrent, progressive, bilateral, or steroid-dependent, can justify a lower threshold for ATT once alternative etiologies have been ruled out. Randomized evidence from an endemic setting suggests ATT can improve outcomes in IGRA-positive uveitis of undetermined cause, but treatment thresholds should remain context-dependent, particularly in low-prevalence regions where incidental IGRA positivity is more common. Ongoing surveillance for TB development remains important in patients with TB infection receiving immunosuppressive therapy, even after LTBI treatment, as preventative therapy was shown to reduce but not eliminate TB risk.

## Data Availability

No datasets were generated or analysed during the current study.
